# Correction: Competing Interests

**DOI:** 10.1186/s42238-024-00254-8

**Published:** 2025-01-06

**Authors:** Journal of Cannabis Research

**Affiliations:** London, UK

**Correction** **:** **Journal of Cannabis Research 6, 25 (2024).**

**https://doi.org/10.1186/s42238-024-00232-0,**

**Journal of Cannabis Research 6, 21 (2024).**

**https://doi.org/10.1186/s42238-024-00226-y,**

**Journal of Cannabis Research 6, 3 (2024).**

**https://doi.org/10.1186/s42238-024-00215-1,**

**Journal of Cannabis Research 5, 10 (2023).**

**https://doi.org/10.1186/s42238-023-00180-1,**

**Journal of Cannabis Research 6, 12 (2024).**

**https://doi.org/10.1186/s42238-024-00221-3,**

**Journal of Cannabis Research 6, 11 (2024).**

**https://doi.org/10.1186/s42238-024-00220-4,**

**Journal of Cannabis Research 4, 55 (2022).**

**https://doi.org/10.1186/s42238-022-00166-5**

After publication of these articles (Fleisher-Berkovich et al. 2024; Caprari et al. 2024; Brooks-Russell et al. 2024; Zolotov et al. 2023; Zaman et al. 2024; Geweda et al. 2024; Aviram et al. 2022), it is reported that the Competing Interests Declaration needs to be updated as follows:

Author NB is an Editor for *Journal of Cannabis Research*. Author NB was not involved in the journal’s review of, or decisions related to this manuscript (Fleisher-Berkovich et al. 2024).

 Author GC is an Editor and Author CC is an Associate Editor for Journal of Cannabis Research. Authors GC and CC were not involved in the journal's review of, or decisions related to this manuscript (Caprari et al. 2024).

Author ABR is an Editor for *Journal of Cannabis Research*. Author ABR was not involved in the journal’s review of, or decisions related to this manuscript (Brooks-Russell et al. 2024).

Author JA is an Editor for *Journal of Cannabis Research*. Author JA was not involved in the journal’s review of, or decisions related to this manuscript (Zolotov et al. 2023)

Author DSH is a member of the editorial board for *Journal of Cannabis Research*. Author DSH was not involved in the journal’s review of, or decisions related to this manuscript (Zaman et al. 2024).

Author MAEl is a member of the editorial board for *Journal of Cannabis Research*. Author MAEl was not involved in the journal’s review of, or decisions related to this manuscript (Geweda et al. 2024).

Author AH is a member of the editorial board for *Journal of Cannabis Research*. Author AH was not involved in the journal’s review of, or decisions related to this manuscript (Aviram et al. 2022).

The original articles (Fleisher-Berkovich et al. 2024; Caprari et al. 2024; Brooks-Russell et al. 2024; Zolotov et al. 2023; Zaman et al. 2024; Geweda et al. 2024; Aviram et al. 2022) has been updated.”

## References

[CR1] Fleisher-Berkovich S, Sharon N, Ventura Y, et al. Selected cannabis cultivars modulate glial activation: in vitro and in vivo studies. J Cannabis Res. 2024;6:25. 10.1186/s42238-024-00232-0. 38778343 10.1186/s42238-024-00232-0 PMC11110427

[CR2] Caprari C, Ferri E, Vandelli MA, et al. An emerging trend in Novel Psychoactive Substances (NPSs): designer THC. J Cannabis Res. 2024;6:21. 10.1186/s42238-024-00226-y. 38702834 10.1186/s42238-024-00226-y PMC11067227

[CR3] Brooks-Russell A, Wrobel J, Brown T, et al. Effects of acute cannabis inhalation on reaction time, decision-making, and memory using a tablet-based application. J Cannabis Res. 2024;6:3. 10.1186/s42238-024-00215-1. 38308382 10.1186/s42238-024-00215-1 PMC10837858

[CR4] Zolotov Y, Lomba J, Ghiroli M, et al. “It doesn’t make any sense to even try”: the disruptive impact of COVID-19’s first wave on people with chronic pain using medical cannabis in New York. J Cannabis Res. 2023;5:10. 10.1186/s42238-023-00180-1. 36978185 10.1186/s42238-023-00180-1 PMC10049907

[CR5] Zaman T, Bravata DM, Byers A, et al. A national study of clinical discussions about cannabis use among Veteran patients prescribed opioids. J Cannabis Res. 2024;6:12. 10.1186/s42238-024-00221-3. 38493111 10.1186/s42238-024-00221-3 PMC10943860

[CR6] Geweda MM, Majumdar CG, Moore MN, et al. Evaluation of dispensaries’ cannabis flowers for accuracy of labeling of cannabinoids content. J Cannabis Res. 2024;6:11. 10.1186/s42238-024-00220-4. 38461280 10.1186/s42238-024-00220-4 PMC10924369

[CR7] Aviram J, Atzmony D, Frenklakh A, et al. THC degradation does not impair the accuracy of THC doses aerosolized by the metered-dose SyqeAir inhaler: a 24-month stability trial. J Cannabis Res. 2022;4:55. 10.1186/s42238-022-00166-5. 36274184 10.1186/s42238-022-00166-5 PMC9590197

